# Assessment of the effect of intraarticular injection of autologous adipose-derived mesenchymal stem cells in osteoarthritic dogs using a double blinded force platform analysis

**DOI:** 10.1186/1746-6148-10-143

**Published:** 2014-07-01

**Authors:** Jose M Vilar, Miguel Batista, Manuel Morales, Angelo Santana, Belén Cuervo, Mónica Rubio, Ramón Cugat, Joaquín Sopena, Jose M Carrillo

**Affiliations:** 1 Department of Animal Pathology, Faculty of Veterinary Medicine, Universidad de Las Palmas de Gran Canaria, Trasmontaña S/N, Arucas, 35413 Las Palmas, Spain; 2 Departamento de Medicina y Cirugía Animal, Universidad CEU Cardenal Herrera, C/Tirant lo Blanc, 7, Alfara del Patriarca, Valencia 46117, Spain; 3 Fundación García Cugat, Madrazo 43, Barcelona 08006, Spain; 4 Instituto de Ciencias Biomédicas Universidad CEU Cardenal Herrera, C/del pozo s/n, Alfara del Patriarca, Valencia 46115, Spain; 5 Garcia Cugat Foundation-UCH Chair, Barcelona, Spain; 6 Artroscopia GC, Hospital Quirón Barcelona, Plaza Alfonso Comí, 12, Barcelona, Spain

**Keywords:** Osteoarthrosis, Hip, Adipose-derived mesenchymal stem cells, MSC, Force platform

## Abstract

**Background:**

Regenerative medicine using Mesenchymal Stem Cells (MSC) alone or combined with Plasma Rich in Growth Factors (PRGF) is a rapidly growing area of clinical research and is currently also being used to treat osteoarthritis (OA). Force platform analysis has been consistently used to verify and quantify the efficacy of different therapeutic strategies for the treatment of OA in dogs including MSC associated to PRGF, but never with AD-MSC alone.

The aim of this study was to use a force platform to measure the efficacy of intraarticular ADMSC administration for limb function improvement in dogs with severe OA.

**Results:**

Ten lame dogs with severe hip OA and a control group of 5 sound dogs were used for this study. Results were statistically analyzed to detect a significant increase in peak vertical force (PVF) and vertical impulse (VI) in treated dogs. Mean values of PVF and VI were significantly improved within the first three months post-treatment in the OA group, increasing 9% and 2.5% body weight, respectively, at day 30. After this, the effect seems to decrease reaching initial values.

**Conclusion:**

Intraarticular ADMSC therapy objectively improved limb function in dogs with hip OA. The duration of maximal effect was less than 3 months.

## Background

Osteoarthritis (OA) is a common degenerative disease in veterinary medicine that affects various tissues surrounding joints such as articular cartilage, subchondral bone, synovial membrane, and ligaments, although cartilage is the main tissue afflicted by OA [1-3]. However, restoration of the diseased articular cartilage in patients with OA is still a challenge for researchers and clinicians. Currently, several therapeutic regenerative strategies have investigated whether autologous mesenchymal stem cells (MSCs) have significant effects on regeneration and/or maintenance of articular cartilage in OA [4-6].

Autologous MSC therapy is based on the isolation of these cells from tissues such as fat or bone marrow [7] and then after culture expansion, they are administered back to the patient [8] on account of the demonstrated affinity that MSCs have for damaged joint tissue, such as cruciate ligaments, menisci, and cartilage [9]. In veterinary medicine, previous studies evaluating autologous ADMSC therapy showed clinical improvement in horses and dogs with orthopedic conditions [10-14]. Recent investigations have shown that growth factors contained in platelet-rich plasma (PRGF) act as vehicles that could act as potentiators or even extend the effect of MSCs [6,15,16]. This may be explained by the antiinflammatory effect of PRGF, which could help the MSCs differentiate in a more favorable environment [3]. At this point, knowing the onset and duration of action MSCs have in canine OA should be highly interesting, as well as further understanding of their efficacy. If previous subjective studies seem to show certain effects, our research hypothesis should be that this effect could be detected and quantified by kinetik devices such as force platforms.The aim of this study was to use force platform kinetic analysis to demonstrate that the effect of a single intraarticular injection of ADMSCs alone is more limited with respect to time, compared with ADMSCs + PRGF in 10 dogs with hip OA. The obtained results were compared with other medical strategies in canine OA and the advantages and disadvantages discussed.

## Results

The body weight of enrolled dogs ranged from 44 to 56 kg (mean ± SD = 50.51 ± 6.76 kg), and ages were 4 to 8 years (5.2 ± 1.7 years). The mean (±SD) value for walking velocity of both sound (control) and diseased groups of dogs was 1.6 ± 0.5 m/s. No significant difference in walking velocity existed between dogs (*P* = 0.06). PVF and VI mean values are summarized in Table 1.

**Table 1:** Mean and standard deviation of PVF and VI (n = 9) in % dog weight (N/N and N.s/N, respectively) applied on the diseased leg

**Day**	**0**	**30**	**90**	**180**
PVF ML	40.14 ± 2.13	49.22 ± 0.961	41.62 ± 1.32	39.73 ± 0.96
PVF LL	51.77 ± 0.79	53.69 ± 0.38	53.25 ± 0.64	51.91 ± 1.22
PVF S	47.40 ± 1.43	47.74 ± 1.34	47.95 ± 1.35	47.94 ± 1.65
VI ML	12.19 ± 0.60	14.76 ± 0.29	12.48 ± 0.4	11.95 ± 0.29
VI LL	15.65 ± 0.31	16.16 ± 0.1	15.98 ± 0.2	15.65 ± 0.38
VI S	14.51 ± 0.47	14.62 ± 0.41	14.67 ± 0.42	14.67 ± 0.51

PVF ML: peak vertical force in the more-lame limbs. PVF LL: peak vertical force in the less-lame limbs. VI ML: vertical impulse in the more-lame limbs VI LL: vertical impulse in the less-lame limbs. PVF S: peak vertical force in the control group. VI S:vertical impulse in the control group.

Data are shown for each day of observation.

### Analysis of PVF

More-lame limbs analysis showed that differences in % PVF between D0 and D30 were significant (p-value < 0.001). Between the other periods, differences were of no significance (p-value > 0.93).

Compared with the control group, % PVF at D0, D90 and D180 is significantly less (p-value < 0.001). In comparison, at D30 difference was non significant (p-value = 0.666).

**Figure 1:**
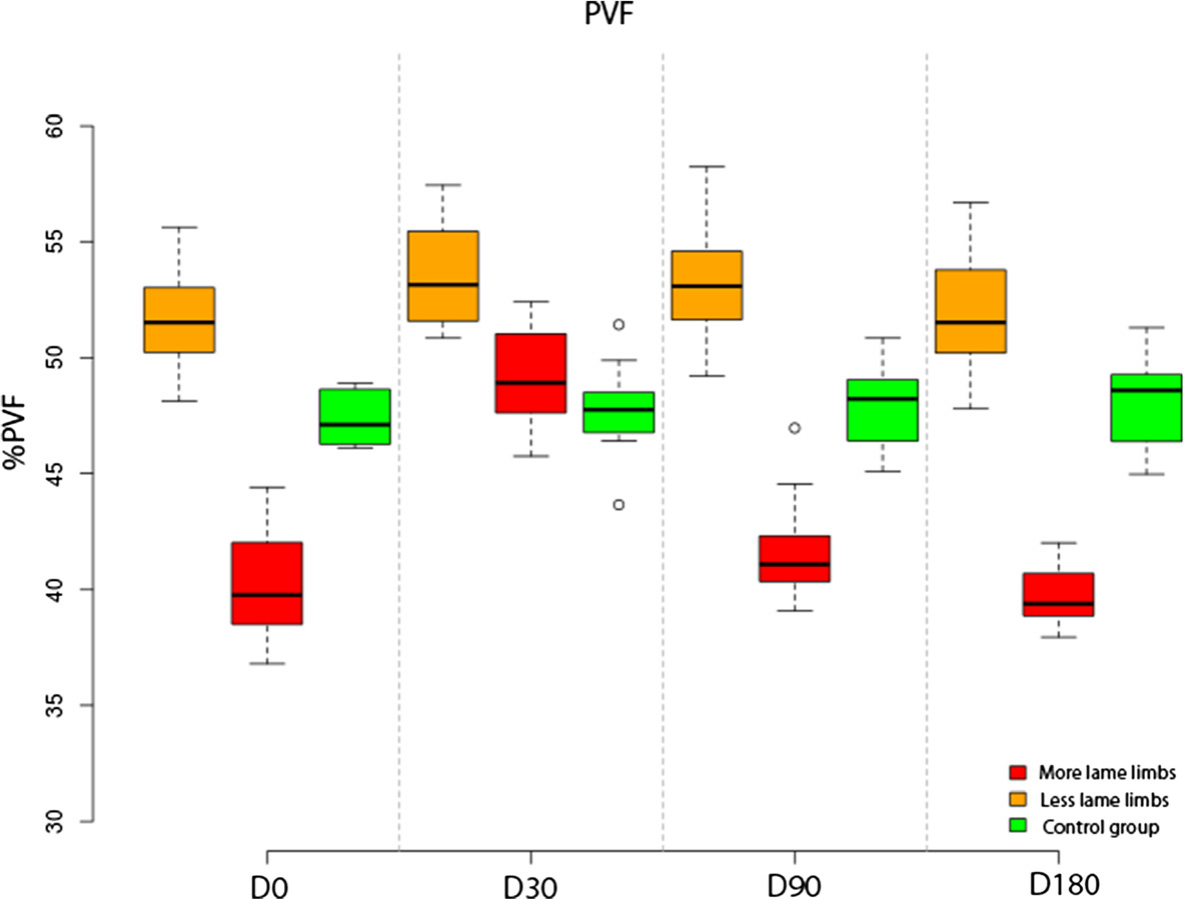
Evolution of PVF in lame group dogs after treatment at the 6-month follow-up period.

Less-lame limbs analysis showed non significant differences at all study periods (p-value < 0.588).Compared with control group, % PVF at D30, D90 and D180 is significantly greater (p-value < 0.001). At D0 this difference was non significant (p-value =0.114) (Figure 1).

### VI analysis

More-lame limbs analysis showed that differences in % VI between D0 and D30 were significant (p-value < 0.001). With the other periods, differences were not significant (p-value > 0.99).

Compared with the control group, % VI at D0, D90 and D180 is significantly less (p-val < 0.001). In comparison, at D30 difference became non significant (p-value > 0.44.

**Figure 2:**
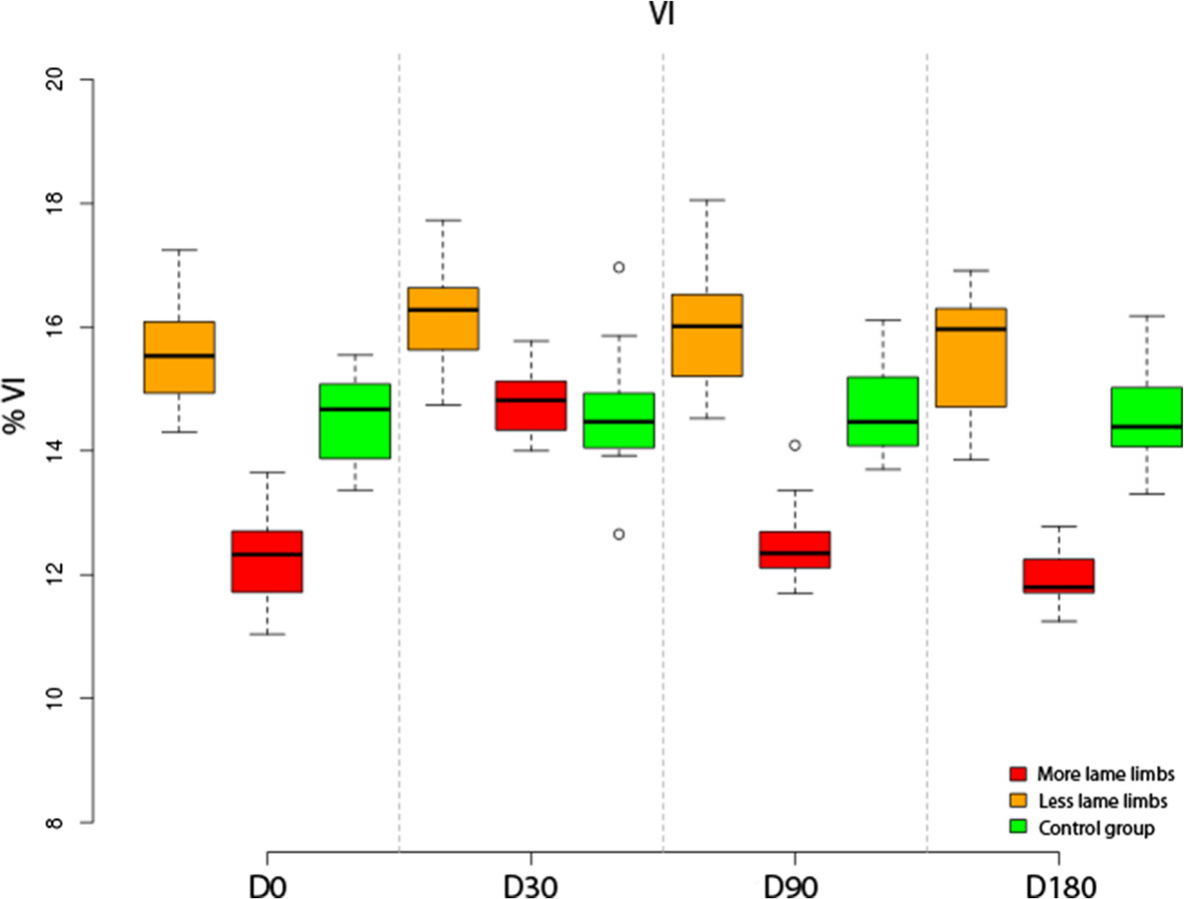
Evolution of VI in lame group dogs after treatment at the 6-month follow-up period.

Less-lame limbs analysis showed no significant differences at any control period (p value = 0.579).Compared with the control group, significant differences at D30 and D90 were found (p-values < 0.001) (Figure 2).

### Association between more and less-lame limbs

The fitted linear mixed model shows a negative association between % PVF in less-lame limb and more-lame limb (β_1_ = 0, p-value = 0.015). In % VI the same was found (β_1_ = 0, p-val = 0.34).

The validity of the model fit was assessed by testing normality and homoscedasticity of the residuals. Both assumptions could be accepted: the Shapiro-Wilk test for normality and Levene’s test for homoscedasticity were not significant (P = 0.79 and P = 0.89, respectively).

## Discussion

The aim of the current study was to test the effectiveness of ADMSCs in lame OA dogs using a force platform. The ground reaction forces-related aspects of the gait, such as the PVF and VI, which represent maximal weight bearing and distribution of forces through time, respectively. Allowing for the objective measurement of the clinical impact of ADMSC treatment on the function of the limb during the stance phase of walking. Force platform analysis could be complemented with kinematic studies since three-dimensional kinematic alterations in hip dysplasia have been well described [17,18].

The lack of a direct relationship between radiographic evidence of OA and force platform findings is well known; in the current study, diseased dogs were selected both on the basis of the presence or absence of radiographic signs of severe OA (D-E degrees of hip dysplasia) and for clear lameness determined by platform gait analysis objectively [19].

Some authors [20,21] reported that force platform gait analysis at trot was much more sensitive than at walk for low-grade hind limb lameness, but not for severe lameness. Based in previous experience [22], in the OA dogs group lameness was evident by direct observation, even at walk.

Although each dog had bilateral lameness, the authors believed that it would only be possible to obtain confident data from the more-lame limbs (lesser PVF), in order to avoid a possible bias caused by inconsistent weight redistribution to the less affected contra-lateral hind limb. In fact, mean values showed how less-lame limbs seemed to perform better than limbs from the control group over the whole study period.

A substantial improvement was observed both in PVF and VI values, but only in the first month post inoculation of ADMSCs; afterwards the animals seemed to worsen returning to the pretreatment condition. Although some authors [6,23-25] found independent evolution of both PVF and VI values, in this study it was showed how these two parameters progressed consistently during the different study periods; the authors believe that this occurred due to the limited effect of the treatment in terms of time; after D30, beyond this point, animals clearly returned to the initial state.

Selection of a control group is determinant to test the efficacy of a treatment for lameness; in fact, when a treated group could improve its lameness, a lame non-treated or placebo control group could worsen, making these animals unable to provide fixed reference data [26]. In accordance with this, the experimental study developed here used a control group of sound dogs that could provide fixed reference data.

Various adipose tissue donor sites have been reported in revised literature: retroperitoneal adipose tissue [27], lateral thoracic area [28], gluteous fat [29] or inguinal region [8,30]. The authors preferred this location to others for its easier access, abundant quantities of fat, absence of surgical complications and production of a non-visible scar.

With regards to the role that other adjuvants associated to ADMSC could play increasing the effect, some results published in 2003 showed that the use of MSCs deposited in a fibrin matrix would be useful [31]. More recently, another study [6] objectively demonstrated how the effect of the association ADMSC + PRGF-Endoret was prolonged beyond six months; however the current study showed evidence that ADMSCs alone have a significant but short effect in terms of time. Based on these results, it is likely that modulation of the matrix or cells will need to be accomplished to observe long-term benefit of MSCs for cartilage repair [3]. Not only could adjuvants play a role in the duration of effect of MSCs therapy; joint idiosyncrasy, and more specifically primary pathologies that cause joint instability such as hip dysplasia or cranial cruciate ligament rupture, they could also noticeably shorten the effect because of the constant reactivation of the associated OA. Presence of side-effects in MSC therapy has also been recently investigated: comparing intra-articular injections of autologous, allogeneic and xenogenic MSCs in horses, only a moderate acute inflammatory response was developed, less evident in autologous cells [5]. In this study, only one dog experimented a transitory worsening after injection. This fact could probably be attributable to incorrect technique in the administration of the cells, because the researcher had to perform various attempts to reach the articular space.

This study objectively demonstrated that a single intraarticular administration of ADMSCs alone decreases pain and lameness in dogs with OA during a period less than three months, at which point PVF and VI values returned to being similar to the pre-treatment status. These results differ with those previously published [6] where improvement was still present after six months of treatment with MSCs associated to PRGF Endoret®. The identical study design suggests that PRGF strongly plays a potentiating role, potentiating the effect of MSC therapy, as shown by other authors [15,16]. In 2007 [13] a multicentre study with MSC alone showed subjective improvement during a 3-month period using different scoring systems, although the results from the current study were supported by objective kinetic data, the different study design (different breeds and conformations, use of a diseased control group) could explain these differences. On the other way, the authors are of the opinion that a follow up of six months could be considered as a standard for testing the evolution of medical or surgical treatments; in fact although the dogs in the current study seemed to improve during the first month after treatment, this effect was reverted before three months after treatment. This fact should induce researchers to objectively determine when a new cycle of treatment should be useful to stop a relapse [32], and clinicians to set out a therapeutic strategy that should be based in MSC associated to PRGF, since a much longer-lasting effect has been demonstrated [6,33].

Regarding statistical analysis, more complex models could have been considered but the authors chose this one because it offers an adequate compromise between complexity and ability to represent the relationships between the considered variables [34,35].

## Conclusion

The measurement of PVF and VI during a follow-up period of six months demonstrated quantitatively that the efficacy of ADMSc therapy alone is significant in terms of improvement of hip joint lameness due to OA, although this effect decreased progressively between D30 and D90.

## Methods

The research protocol was revised and authorized by the Ethics Committee of Animal Welfare (CEBA) at the University of Las Palmas de Gran Canaria (Spain) with reference code: 001/2010 CEBAULPGC.

### Animals

Nine adult client-owned Canarian Presa dogs (5 males, 4 females) with lameness and pain attributed to OA associated with hip dysplasia were included in the study (one of the ten initially selected was finally discarded because it was lost to follow up). The dogs were affected by chronic OA and had not received any kind of medication (e.g., non-steroidal anti-inflammatory drugs, analgesics), nutraceuticals (e.g., glucosamine or chondroitin, vitamin E, omega-3 fish oil), or adjunctive therapies (e.g., acupuncture) for at least 2 months. A control group consisted of 5 sound and healthy dogs of the same breed.

None of the dogs were forced to perform physical activity. Dog owners were informed and granted a signed consent for the whole procedure.

Ventrodorsal radiographs were performed under sedation with dexmedetomidine 0.05 mg IV (dexdomitor, Esteve, Barcelona, Spain) and analgesia with butorfanol 0.05 mg/kg (torbugesic, Pfizer, Madrid, Spain). The obtained images confirmed the presence of OA compatible with D and E degrees of hip dysplasia as defined by the Fédération Cynologique Internationale (World Canine Organization).D- degree dysplasic dogs showed obvious deviation from the normal with evidence of a shallow acetabulum, flattened femoral head, poor joint congruency, and in some cases, subluxation with marked changes of the femoral head and neck. E-degree dysplasic dogs showed complete dislocation of the hip and severe flattening of the acetabulum and femoral head [36]. Additional radiographs of knee and elbow joints and lumbosacral region were taken after physical, orthopedic and neurologic examinations were performed to ensure that hip OA was the main reason for the observed clinical signs and that general health was otherwise normal.

### Extraction and culture of ADMSCs

Stem cell extraction phase was performed under premedication with a combination of buprenorfine 0.01 mg/kg IM (buprex, RB Pharmaceuticals, Bogotá, Colombia) and acepromazine 0.05 mg/kg IM (equipromacina, Fatro ibérica, Barcelona, Spain); general anesthesia was induced with propofol 3 mg/kg (vetofol, Esteve, Barcelona, Spain) and maintained with sevofluorane (sevoflo, Abbott, Madrid, Spain). Patients were positioned in decubitus supinus. A biopsy of 20 g of subcutaneous fat tissue (4–5 cm^3^) was collected from the inguinal region through a small surgical incision, and 120 ml of blood was isolated under aseptic conditions and processed with the DogStem® kit according to manufacturer’s instructions. The incision was sutured with a simple, interrupted pattern. Meloxicam 0.1 mg/kg/24 h PO (metacam, Boehringer ingelheim, Barcelona, Spain) was administered during 3 days post-surgery.

Immediately after sample collection, fat biopsy and blood (in anti-coagulant container) were sent at 4°C for cell isolation and amplification under current GMP conditions to the Fat-Stem Laboratory (Belgium). The fat was processed with colagenase and by centrifugation the cells were concentrated; the cells were cultured in a biorreactor with controlled temperature as well as O_2_ and CO_2_ concentration. Quality control was based in cell markers, sterility tests and viability counts. Two weeks after biopsy the Fat-Stem Laboratory returned the cultivated cells in two 2 ml certified tubes containing 15 million adipose mesenchymal stem cells per tube.

### Inoculation of ADMSCs

Once the ADMSCs were received they were infiltrated aseptically into the hip joints through conventional arthrocentesis sites. For this phase the dogs were previously sedated with the same protocol used to take the radiographs.

The needle was introduced just cranioproximal to the trochanter major, aimed slightly ventrally and caudally. The appearance of joint fluid confirmed proper needle placement [37]. Once the excessive synovial liquid was drained, the AMSCs were injected. Owners were advised to use meloxicam, if needed.

### Gait analysis

Gait analysis was performed using a single platform mounted in the center of, and level with, a 7-m runway covered by a rubber mat. The mat weight was discarded setting to “0 force” with the tare button after the platform was covered. Dogs were leash guided at walk over the force platform by the same handler. Walk velocity was measured by use of a motion sensor (Pasco, California, USA) positioned 1 m from the platform. This device allowed the handler to ensure that animals walked homogeneously in a narrow interval of velocity (1.6 ± 0.5 m/s) and acceleration (≤ ± 0.5 m/s^2^).

Five valid trials, at a sampling frequency of 250 Hz, were obtained for each dog by a blind researcher (JMV). A trial was considered valid when the limb fully contacted the force platform, and with the dog walking next to the handler without pulling on the leash. The trial was discarded if the dog was distracted during the measurement, if the limb struck the edge of the force plate, or if any portion of the contralateral paw hit the force plate. A member of the research team (BC) evaluated the trial to confirm which limb touched the center of the force platform.

The platform was interfaced with a dedicated computer using DataStudio (Pasco, California, USA), software specially designed for the acquisition, numerical conversion, and storage of data. A team member (JMV) recorded data from both affected limbs at day 0, 30, 90, and 180 post-treatment; the obtained PVF and VI values were normalized relative to body weight (%) to characterize the possible improvement of lameness during treatment with MSCs.

### Statistical analysis

Parameters in this model were estimated by using the package nlme in the R statistical software [38].

Data were analyzed by a different, blinded researcher (AS) who did not perform acquisition of data.

For the analysis of these data, a linear mixed effects model for a blocked design with repeated measures was considered [34,35]. The experimental factor (time) and the status (lame-sound) of the dog were considered as fixed effects factors, while the blocking factor (dog) was a random effects factor. Because the dogs represent a random sample of the population of interest, any interaction terms modeling differences between dogs in its response when changing from different observation periods will also be expressed as random effects. Thus, the model considered is of the form:

$$y_{\text{ijkl}}=\beta_{i}+\gamma_{j}+{\left(\beta \gamma \right)}_{\text{ij}}+b_{jk_{j}}+b_{\text{ij}k_{j}}+\epsilon_{\text{ij}k_{j}l}$$

with *i* = 0,1,3,6 (months), *j* = 0 (sound), 1 (lame), *k*_*0*_ = 1,…,8, *k*_*1*_ = 1,…,5, *l* = 1,…,5. In this model, *β*_*i*_ represents the effect of time, γ_j_ the effect of the dog being sound or lame and (*βγ*)_ij_ the interaction between these factors. The term *b*_jk_represents the random effects of the dogs, and the *b*_ijk_ represents the random interaction terms between dogs and time, being:

$$b_{jk_{j}}∼N\left(0,\sigma_{1}^{2}\right),\;b_{\text{ij}k_{j}}∼N\left(0,\sigma_{i}^{2}\right),\;\epsilon_{\text{ij}k_{j}l}∼N\left(0,\sigma^{2}\right)$$

Significance of the differences in PVF and VI between periods of observation were tested by means of analysis of variance of these models. Following this analysis, post-hoc comparisons between fixed effects were performed using Tukey’s procedure. For assessing the validity of the model, the Shapiro-Wilk test was applied for testing normality of the residuals.

For assessing the relationships between supporting force in the more-lame and the less-lame limbs and also between vertical impulse in the two limbs, a regression model with random effects of dog on slope and intercept was used:

$$y_{\text{ij}}=\beta_{0}+b_{0}+\left(\beta_{1}+b_{1}\right)x_{i}+\epsilon_{\text{ij}}$$

with:

$$b_{0}∼N\left(0,\sigma_{0}^{2}\right),\;b_{1}∼N\left(0,\sigma_{1}^{2}\right),\;\epsilon_{\text{ij}}∼N\left(0,\sigma^{2}\right)$$

Here y_ij_ represents the value (PVF or VI) in the less-lame limb and x_ij_ the value in the more-lame limb.

Significance level was set at *P* ≤ 0.05 in all tests.

## Abbreviations

ADMSCs: Adipose-derived mesenchymal stem cells; MSCs: Mesenchymal stem cells; OA: Osteoarthritis; PVF: Peak vertical force; VI: Vertical impulse; N: Newton.

## Competing interests

The authors declare that they have no competing interests.

## Authors’ contributions

JMV, MB, MR and JMC designed the study, drafted the manuscript, and analyzed data; RC and JS revised and edited the manuscript; AS designed and developed the statistical analysis; MM and BC performed the selection of animals and helped with the revision of the manuscript. JMV and AS were blind researchers. All authors read and approved the final manuscript.
